# Cowpeas in Northern Ghana and the Factors that Predict Caregivers’ Intention to Give Them to Schoolchildren

**DOI:** 10.1371/journal.pone.0072087

**Published:** 2013-08-12

**Authors:** Abdul-Razak Abizari, Nerisa Pilime, Margaret Armar-Klemesu, Inge D. Brouwer

**Affiliations:** 1 Division of Human Nutrition, Wageningen University, Wageningen, The Netherlands; 2 Department of Community Nutrition, School of Medicine and Health Sciences, University for Development Studies, Tamale, Ghana; 3 Department of Nutrition, Noguchi Memorial Institute for Medical Research, University of Ghana, Legon, Ghana; Tulane University School of Public Health and Tropical Medicine, United States of America

## Abstract

**Background:**

Cowpeas are important staple legumes among the rural poor in northern Ghana. Our objectives were to assess the iron and zinc content of cowpea landraces and identify factors that predict the intention of mothers/caregivers to give cowpeas to their schoolchildren.

**Methods and Findings:**

We performed biochemical analysis on 14 landraces of cowpeas and assessed the opinion of 120 caregiver-child pairs on constructs based on the combined model of the Theory of Planned Behaviour and Health Belief Model. We used correlations and multiple regressions to measure simple associations between constructs and identify predictive constructs. Cowpea landraces contained iron and zinc in the range of 4.9–8.2 mg/100 g d.w and 2.7–4.1 mg/100 g d.w respectively. The landraces also contained high amounts of phytate (477–1110 mg/100 g d.w) and polyphenol (327–1055 mg/100 g d.w). Intention of mothers was strongly associated (*r_s_ = *0.72, *P<*0.001) with and predicted (β = 0.63, *P*<0.001) behaviour. The constructs, barriers (β = –0.42, *P* = 0.001) and attitudes towards behaviour (β = 0.25, *P*<0.028), significantly predicted intention albeit the predictive ability of the model was weak.

**Conclusions:**

We conclude that some cowpea landraces from northern Ghana have appreciable amounts of iron and zinc but probably with poor bioavailability. Attitudes towards giving cowpeas and perception of barriers are important predictors of caregivers’ intention to give cowpeas to their schoolchildren. Finally our results suggest that increasing knowledge on nutritional benefits of cowpeas may increase health values caregivers hold for their children in support of giving cowpeas to schoolchildren.

## Introduction

Iron-deficiency is of public health significance in developing countries [1] and likely to co-exist with zinc deficiencies [2], [3]. In northern Ghana, more than two-thirds of schoolchildren are likely to suffer from iron-deficiency [4]. Long term consequences include decreased physical work capacity and future productivity [5], [6]. An integration of approaches has been proposed as key to the reduction in prevalence of iron-deficiency [7], [8]. Strategies to fight iron-deficiency need to be culturally sensitive, acceptable and carefully linked with the food culture of communities. One of such strategies is the promotion of the consumption of local staple foods that are rich in iron. However local staples in settings like northern Ghana are largely cereals, roots and tubers, and legumes, which often do not contain high native iron. Therefore targeted breeding for higher concentration of native iron (biofortification) has been proposed as a sustainable intervention to increase dietary iron intake [9]. Early in the steps towards such biofortification is the identification of varieties with high native iron and/or zinc to serve as breeding parents [10].

Being one of the widely consumed staple crops in Ghana, cowpeas (*Vigna unguiculata* (L.) Walp) have received attention as a candidate crop for biofortification to improve its native iron as well as zinc concentration. Cowpeas are native to northern Ghana [11]. They have high nutritional significance due to their good quality protein content and significant amounts of vitamins and minerals like iron and zinc [12], [13]. Over the years cowpeas have grown from being regarded as “poor man’s meat” [14] to one that is consumed across socio-economic strata. Therefore cowpeas may have the potential to contribute to better iron and nutritional status.

Even though cowpeas are already an intricate part of the food culture of northern Ghana [13], it is not known what factors influence mothers to give cowpeas to their schoolchildren. Understanding the significant factors that predict cowpea consumption can provide important insight for the development of effective interventions leading to increased cowpea consumption not only within a school feeding programme but also at the household.

To date, two popular psychosocial theories (Theory of Planned Behaviour (TPB) and Health Belief Model (HBM)) have found wide use in explaining influential variables in food-related behaviours. According to the TPB, behaviour is a conscious act proximally mediated by intention [15]. The HBM on the other hand posits that a health behaviour results from a set of core beliefs [16]. It has been proposed that a combination of these two complementary theories will help broaden our understanding of factors that influence dietary behaviour [17], [18].

In this paper, as part of the early steps in identifying cowpeas with potential for biofortification, we assessed the iron and zinc content of available cowpea landraces in northern Ghana. Secondly, using a combined model as proposed by Sun et al. [17], we aimed at identifying factors that influence the intention of mothers/caregivers to give cowpeas to their schoolchildren.

## Materials and Methods

### Study Area

This study was conducted in the Tolon-Kumbungu district of the Northern Region of Ghana. The region is well-suited for cowpea production and Tolon-Kumbungu district is among the three top production-processing-consuming sites for cowpea in the region [19]. The research area is located within the Guinea Savannah agro-ecological zone and has two distinct seasons; rainy season (April – September) and a dry season (December – March) characterized by relatively high day temperatures (35–40°C). The people of this area are largely subsistence farmers [20]. Two communities, Kpaligung and Tibung, were purposively selected because they were participating in a larger nutrition study that sought to investigate the potential role of cowpeas in improving iron status through school feeding programme. At the time, these two communities were the only ones piloting the government-supported school feeding programme in the district.

### Study Design and Subjects

#### Cowpea landrace study

Key informant interviews with 3 farmers and 3 market women were conducted in March 2008 to identify locally available landraces of cowpeas in the selected communities and the largest market in the main city, Tamale. Landraces were identified based on the local knowledge and experience of local farmers and market women. For each landrace, a sample of 200 g was collected after cleaning and separating mixed landraces. Seeds with holes or weevil attack were removed by hand. All collected samples were kept in transparent polythene bags and labelled with their corresponding local names as given by the key informants. The samples were sent to Wageningen University, The Netherlands, and stored at −20°C pending analysis.

#### Cowpea acceptability study

This was a cross-sectional study conducted in two schools within two communities (Kpalgun and Tibung) in November 2008. In each school 60 schoolchildren (6–12 years) in lower primary (classes 1–3) were randomly selected to participate, a sample size assumed to be adequate in research based on the TPB [21]. The corresponding 120 caregivers of the selected children were invited for interview and none of them declined participation. In each of the two schools, children were randomly selected from a sampling frame of pupils in lower primary (classes 1–3). The sampling frame was constructed separately for each school by pooling the class registers of lower primary. Inclusion of children in the sampling frame was independent of whether they were present in or absent from school on the day of sampling frame construction. If two or more children were selected from one household, one of them was randomly selected by lottery to participate in the study. The study was approved by the Institutional Review Board of Noguchi Memorial Institute for Medical Research, University of Ghana (NMIMR-IRB 022/08-09). Each volunteer gave verbal informed consent prior to participation.

### Questionnaire Development

**Figure 1:**
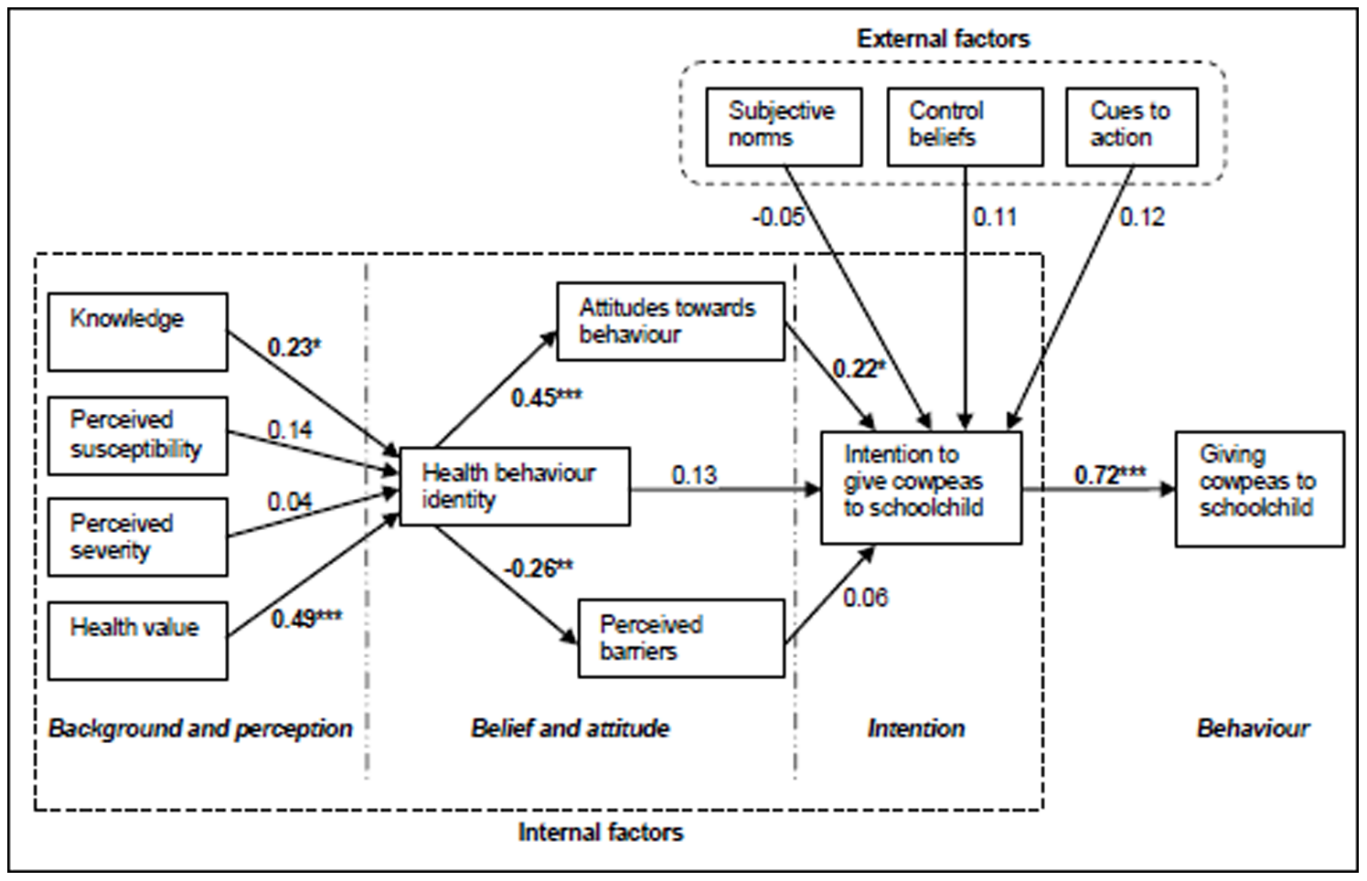
A combined model of the Theory of Planned Behaviour and Health Belief Model with correlation coefficients between related constructs. Adapted from Sun et al. [17]. *P<0.05; **P<0.01; ***P<0.001 (2-tailed).

An 89-item (grouped into 12 constructs, see **Table 1**) research questionnaire was developed along the recommendations of Francis et al [21] and based on the Theory of Planned Behaviour [15] and the Health Belief Model [16]. These two models were combined into the study model as described by Sun et al [17], **Figure 1**. Relative to behaviour, constructs were grouped into internal and external factors. The internal factors were further grouped into: background and perception, belief and attitude, and intention. The construct subjective norm, left out of Sun et al.’s model, was added to the study model because in an African setting the values of extended family and community significantly influence behaviour of an individual [22]. The items included in each construct were drawn from previous studies [17], [23] and literature review on cowpea attributes from West African countries (Nigeria, Ghana and Senegal) [24]–[29]. The questionnaire also included questions concerning background information of the respondents and their schoolchildren. The items in the 12 constructs were verified in a focus group discussion and pre-tested in a site similar to the study site. Where applicable, constructs were modified to suite local knowledge and practice. The questionnaire was translated into the local language (*Dagbani*) and administered by well-trained research assistants who were familiar with the research area and spoke the local language.

**Table 1:** Operational definition of constructs used to examine factors that predict intention of caregivers to give cowpeas to their schoolchildren.

Construct	Operational definition
Knowledge	The caregiver’s knowledge about the relationship between cowpeas and health, and specifically to malnutrition and iron-deficiency anaemia
Perceived susceptibility	The caregiver’s subjective perception of her schoolchild being malnourished and anaemic
Perceived severity	The caregiver’s feelings concerning the seriousness of her schoolchild being malnourished and anaemic
Health value	The importance caregiver places on the consequences of her schoolchild being malnourished and anaemic
Health behaviour identity	The caregiver’s opinion of the expected consequence of giving cowpeas to the schoolchild
Attitudes towards behaviour	Favourable or unfavourable disposition of the caregiver towards giving cowpeas to the schoolchild
Perceived barriers	The caregiver’s beliefs about costs or negative aspects of cowpea consumption by the schoolchild
Cues to action	Triggers that stimulate the caregiver to give cowpeas to her school child.
Subjective norms	The caregiver’s perceived social pressure to give or not to give the schoolchild cowpeas (who is important for the behaviour and is the opinion of that person important?)
External control belief	The caregiver’s perceived presence of factors that may facilitate or impede giving cowpeas to the schoolchild
Behavioural intention	The caregiver’s readiness to give cowpeas to the schoolchild
Behaviour	Giving cowpeas to the schoolchild

### Scale Measurements and Analysis

Individual items, phrased as statements, of each construct (except Intention and Behaviour) were rated on 5-point Likert [30] response options: strongly disagree, disagree, neutral (neither disagree nor agree), agree and strongly agree; recoded as unipolar (+1 to +5) or bipolar (–2 to +2) depending on the nature of the question. The score for each construct was computed as the sum of individual item scores. The scores for the constructs *“Attitudes towards behaviour”* and *“Subjective norms”* were sums of products of paired items; *attitudes*×*evaluation of attitudes*, and *normative beliefs*×*motivation to comply*, respectively. To show negative, neutral or positive influences, item scores of *attitudes* and *normative beliefs* ranged from –2 to 2 and the scores of the *evaluation of attitudes* and *motivation to comply* ranged from +1 to +5. This resulted in a paired-item score range of –10 to 10. For intention and behaviour, scores were based on the number of times caregivers intended to or had given cowpeas to their schoolchildren in the succeeding or preceding month respectively. Intention was considered high if it was greater than the median intention score of the group (10 times per month) and low if it was equal to or lower than the median score.

### Cowpea Chemical Analysis

Iron and zinc concentration of cowpeas were measured using inductively coupled plasma atomic emission spectrophotometer (ICP-AES, Varian Vista-Pro, Palo Alto, CA, USA) after microwave digestion with a mixture of hydroflouric and nitric acids (HNO_3_-HF-H_2_O_2_). Analytical variation was ∼6% for both iron and zinc. Phytic acid determination was done using a modified Makower method [31] in which the inorganic phosphate liberated from the phytic acid degradation is measured according to the van Veldhoven’s method [32] and expressed as inositol hexaphosphate (IP6). A modified Folin-Ciocalteau method [33] was used to measure total polyphenol concentration of the cowpea seeds.

### Calculation of Phytate-to-mineral Molar Ratios

The respective phytate-to-iron and phytate-to-zinc molar ratios for each landrace were calculated as: phytate content of cowpea (mg)⋅660^–1^/iron content of cowpea (mg)⋅56^–1^; phytate content of cowpea (mg)⋅660^–1^/zinc content of cowpea (mg)⋅65.4^–1^ respectively, where 660, 56 and 65.4 are the molecular weights of phytate, iron and zinc respectively [34], [35].

### Statistical Analysis

Data processing and analysis was done in SPSS software (version 18.0, Armonk, NY, USA). Descriptive statistics were used to examine background characteristics of study participants, constructs and cowpea landraces. Student’s t-test for independent samples was used to compare the difference in chemical composition between white and coloured landraces of cowpeas. Cronbach’s alpha was computed as measure of reliability for each construct. A construct was reliable if Cronbach’s alpha was >0.7 [36]. The corrected item-total correlation of all items in a construct was set at 0.30 [36]. When the item-total correlation was lower than 0.30 the item was deleted from the construct. As such, a total of 7 items were deleted from two constructs; one item from *cues to action* and 6 items from *attitudes towards behaviour*.

Spearman correlations were computed to determine association between related constructs. For constructs that influenced intention, the Mann-Whitney-U test was used to compare whether subjects with a high intention to consume cowpea scored significantly different on any of the constructs from subjects with a low intention. The Wilcoxon signed rank test was used to test the differences in the scores of behaviour and intention.

Four multiple linear regression models were used to determine the relative importance of the predicting constructs for the following outcomes: health behaviour identity, intention and behaviour. All models were controlled for background characteristics of caregivers. Significance value for all tests was set at 0.05 (2-tailed).

Model 1: Health behaviour identity = *f* (Knowledge, Susceptibility, Severity and Value)

Model 2: Intention = *f* (Barrier, Health behaviour identity, and Attitudes towards behaviour)

Model 3: Intention = *f* (Subjective norms, Control beliefs, and Cues to action)

Model 4: Behaviour = *f* (Barrier, Intention)

## Results

### Iron, Zinc, Phytate and Polyphenol Concentrations of Cowpea Landraces

A total of 14 landraces were identified as common landraces from the two communities and the central market. Iron and zinc concentrations ranged from 4.9–8.2 mg/100 g d.w and 2.7–4.1 mg/100 g d.w respectively. Phytate and polyphenol concentrations ranged from 477–1110 mg/100 g d.w and 327–1055 mg/100 g d.w respectively (**Table 2**). With respect to colour of the cowpeas, there was no significant difference (*P*>0.05) in iron, zinc and phytate concentrations between the white and coloured landraces. Molar ratios of phytate-to-iron also did not differ between white and coloured landraces (*P*>0.05). Coloured landraces however had significantly higher concentrations of polyphenols and significantly larger (*P*<0.05) phytate-to-zinc molar ratios (**Table 3**).

**Table 2:** Iron, zinc, phytate and polyphenol composition of landraces of cowpeas locally available in northern Ghana.

Local name	Colour	Iron	Zinc	Phytate^1^	Polyphenols^2^
		mg/100 g dry weight			
*Dagban tuya*	white	6.8	3.4	477	445
*Apagaba ala1*	white	8.2	3.9	519	481
*Komtuya*	white	5.8	4.1	679	335
*Black eye*	white	6.2	3.5	745	385
*Tuchicherigu*	black &white	5.5	3.0	888	327
*Apagaba ala2*	white	5.7	2.7	487	368
*Gampabgi 1*	brown	6.3	3.7	1110	744
*Sanzi sabinli*	brown	7.0	3.3	664	621
*Gampabgi 2*	black	6.2	3.5	561	662
*Milo*	brown	6.5	3.1	610	NA
*Yaminu*	red	5.4	2.7	537	1055
*Sanzi zie*	red	7.7	4.1	895	942
*Brown eye*	white	5.8	3.7	605	NA
*Marfu tuya*	white	4.9	3.6	NA^3^	NA

1 Inositol hexaphosphate (IP6).

2 Gallic acid equivalent (GAE).

3 Not analysed due to insufficient sample.

**Table 3:** Comparisons of iron, zinc, phytate, polyphenol and phytate-to-mineral composition of white and coloured landraces of cowpeas locally available in northern Ghana.

Parameter	Colour of cowpea		*P*
	White	Coloured	
Iron	6.2±1.1 (7)^1^	6.4±0.8 (7)	0.738
Zinc	3.6±0.4 (7)	3.3±0.5 (7)	0.398
Phytate (PA)	570±108 (7)	752±215 (7)	0.077
Polyphenols	403±59 (5)	725±257 (6)	0.023
PA : Iron^2^	8.0±2.1 (6)	10.1±3.0 (7)	0.171
PA : Zinc^2^	16.5±2.9 (6)	22.3±5.4 (7)	0.037

1 Values are mean ± SD (number of landraces).

2 Molar ratio.

### Background Characteristics

More than 50% of the children in school were male. Ages of the school children ranged from 6–12 years with about one-third in the age groups 8–9 years and 10–11 years. Majority (61%) of households indicated that they had more than 10 people in their households. More than 50% of the caregivers of the school children were older than 35 years, 61% of them were mothers and 62% were in polygamous marriage. Only 4% of the caregivers were literate and more than 70% of them were either engaged in farming or trading as their main economic activity (**Table 4**).

**Table 4:** Background characteristics of schoolchildren and their caregivers in northern Ghana.

Characteristic	*n* (Percentage)
*n*	120
Sex of child, male	69 (57.5)
Age of child, years	
6–7	34 (28.3)
8–9	42 (35.0)
10–11	41 (34.2)
≥12	3 (2.5)
Household size	
3–6	11 (9.2)
7–10	36 (30.0)
>10	73 (60.8)
Age of caregiver, years	
19–34	54 (45.0)
35–49	36 (30.0)
>49	30 (25.0)
Relationship of caregiver to child	
Mother	73 (60.8)
Stepmother	10 (8.3)
Grandmother	22 (18.3)
Other relation	15 (11.7)
Marital status of caregiver	
Married (monogamous)	32 (26.7)
Married (polygamous)	74 (61.7)
Widowed/divorced	14 (11.6)
Education of caregiver	
% illiterate	115 (95.8)
Occupation of caregiver	
Farming	38 (31.7)
Trading	53 (44.2)
Housewife	25 (20.8)
Other	4 (3.4)

### Knowledge, Attitude and Perceptions of Caregivers about Cowpeas and Giving Cowpeas to Schoolchildren

Ninety-two percent (92%) of caregivers had the intention to give cowpeas to their schoolchildren at least once per week within the referent month while 82% indicated that they had given cowpeas to their schoolchildren at least once per week within the referent month. Almost all the caregivers agreed that cowpeas contain iron (94%), can prevent iron-deficiency (97%) and support growth (97%) of their schoolchildren. They think of cowpeas as a food that is nutritious (98%), traditional (97%) and tasty (99%), and adds variety (97%) to the diet of their schoolchildren. More than half (57%) of the caregivers however think that cowpeas are not easily digested by their schoolchildren and leaves them feeling uneasy. Nevertheless 97% of them said their schoolchildren like to eat cowpeas. Generally the caregivers agreed that availability on the market (73%), prices (85%), time required to cook cowpeas (71%), weevils (70%), high prices (80%) and preservation (81%) were barriers to giving cowpeas to their school children. In line with their health-related opinions about cowpeas, 70% of the caregivers indicated that “illness” serves as a cue for them to give cowpeas to their schoolchildren.

### Reliability of Constructs and their Correlations

Reliability (Cronbach’s α) of the multiple item constructs ranged from (0.67–0.88). Except for the construct *susceptibility*, the reliability of all other constructs was ≥0.80. Two of the four constructs classified as “background and perception” were significantly correlated with *health behaviour identity*; *knowledge (r_s_* = 0.23, *P = *0.013) and *health value* (*r_s_* = 0.49, *P<*0.001). Within the “belief and attitude” group of constructs, *attitude towards behaviour* (*r_s_* = 0.45, *P<*0.001) and *perceived barriers* (*r_s_* = 0.26, *P = *0.004) showed significant correlations with *health behaviour identity*. *Attitude towards behaviour correlated* (*r_s_* = 0.22, *P = *0.019) significantly with *intention*. None of the three constructs classified as “external factors” significantly correlated with caregivers’ intention to give cowpeas to schoolchildren. Intention to give cowpeas to schoolchildren was positively and strongly correlated (*r_s_* = 0.72, *P<*0.001) with the behaviour of giving cowpeas to schoolchildren (**Table 5**).

**Table 5:** Sample item statements, number of items, reliability and summary values of the constructs from the combined model of the Theory of Planned Behaviour and the Health Belief Model.

Construct	Example of item statement	Numberof items	Cronbach’s α	Median score(IQR)	Range of values
Knowledge	Cowpeas are a blood giving food	11	0.84	45 (44, 49)	11–55
Susceptibility	My schoolchild easily becomes sick	4	0.67	12 (10, 16)	4–20
Severity	Shortage of blood causes poor growth of my schoolchild	7	0.87	28 (24.3, 29)	7–35
Health value	The health of my schoolchild is very important to me	7	0.80	31 (29, 33)	7–35
Health behaviour identity	Giving cowpeas is one of the best things I can do for my schoolchild	2	0.80	8 (8, 9)	2–10
Barriers	I worry about the availability of cowpeas on the market	16	0.85	38.5 (32.3, 45.8)	16–80
Control beliefs	I am the one who decides to give my school child cowpeas	1	–	4 (4, 4.8)	1–5
Cues to action	Important ceremonies like weddings or funerals make my schoolchild want to eat	5	0.81	16 (14, 22)	5–25
Attitudes towards behaviour	(Cowpeas have a good taste)×(my schoolchild prefersfoods that taste good)	16	0.88	38 (32, 47)	−160–160
Subjective norms	(My mother-in-law advices me to give cowpeas to my schoolchild)×(the opinion of my mother-in-law isimportant to me)	14	0.83	−4.5 (−20, 15.5)	−140–140
Behavioural intention	How many times do you intend to give cowpeas to your schoolchild in the coming month	1	–	10 (5, 15)	0–30
Behaviour	How many times have you given cowpeas to yourschoolchild last month	1	–	8 (4, 12)	0–30

Since *attitudes towards behaviour* correlated significantly with *intention*, we checked and found that scores were higher for the high intenders but did not significantly differ the scores of low intenders (z = –0.64, *P = *0.52). All median scores of the items within *attitudes towards behaviour* were positive for both the low and high intenders. Paired comparisons between *intention* and *behaviour* showed that intention to give cowpeas was significantly higher than the behaviour of giving cowpeas (z = –3.177, *P = *0.001). Of the 120 caregivers interviewed the intention-behaviour paired observations were: intention>behaviour, *n* = 62; intention<behaviour, *n* = 24; intention = behaviour, *n* = 34.

### Predicting Health behaviour Identity, Intention and Behaviour

The relative contribution of the predictor variables to the outcome variables for models 1–4 are shown in **Table 6**. Model 1 explained 36% of the variance in *health behaviour identity* and the constructs *knowledge* (β = 0.20, *P = *0.030) and *health values* (β = 0.49, *P*<0.001) significantly predicted health behaviour identity. Model 2 explained only 8% of the variance in *intention* and the constructs *barriers* (β = –0.42, *P = *0.001) and *attitudes towards behaviour* (β = 0.25, *P*<0.028) significantly predicted *intention*. In model 3, none of the external factors significantly explained intention. Model 4 accounted for 40% of the variance in behaviour and intention significantly predicted behaviour (β = 0.63, *P*<0.001).

**Table 6:** Constructs predicting health behaviour identity associated with cowpeas, intention to give cowpeas and giving cowpeas to schoolchildren in northern Ghana^1^.

Model description	Standardized *β*	*P*	R^2^	Adjusted R^2^
Model 1				
Y = Identity			0.43	0.36
Predictors				
Knowledge	0.20	0.030		
Susceptibility	0.02	0.847		
Severity	−0.04	0.652		
Values	0.49	<0.001		
Model 2				
Y = Intention to consume cowpeas			0.17	0.08
Predictors				
Identity	0.06	0.611		
Barriers	−0.42	0.001		
Attitudes	0.25	0.028		
Model 3				
Y = Intention to consume cowpeas			0.07	−0.03
Predictors				
Control	−0.09	0.334		
Cues	−0.001	0.994		
Subjective norms	−0.05	0.637		
Model 4				
Y = Consumption of cowpeas			0.46	0.40
Predictors				
Intention	0.63	<0.001		
Barriers	0.07	0.469		

1 All models were controlled for community, interviewer, caregiver and child characteristics.

## Discussion

### Cowpea Landraces

Our first objective was to identify the cowpea landrace (s) that would be most suitable to promote as source of bioavailable iron and zinc. We found that the locally available landraces contained appreciable amount of iron and zinc but also contained high concentrations of phytate and polyphenols.

The range of values we observed for iron (4.9–8.2 mg/100 g d.w) and zinc (2.7–4.1 mg/100 g d.w) were somewhat lower than the 5.6–10.4 mg/100 g d.w and 3.7–5.4 mg/100 g d.w respectively observed among cowpea landraces in Benin [37]. Based on iron and zinc concentrations the results suggest that *zanzi zee* could be promoted as the most suitable landrace with the potential to improve iron intake. However, the phytate and polyphenol concentrations of the landraces were high but within range of values reported by Madode et al. [37]. Iron and zinc absorption are partly influenced by phytate and polyphenol concentration [35], [38].

A proxy measure of iron and zinc bioavailabilty is the molar ratio of iron-to-phytate and zinc-to-phytate respectively [34], [35], [39], [40]. Iron-to-phytate and zinc-to-phytate molar ratios of <1 and ≤15 respectively are considered predictive of iron [40], [41] and zinc [34], [35] bioavailabilty. As such, all the landraces have low bioavailable iron. For zinc however, *apagaba ala-1* and *dagban tuya* are likely to contain zinc with higher bioavalability. Abizari et al [42] found that iron bioavailability in cowpeas was <2% and their results suggested that rather than polyphenols, phytate-to-iron molar ratio may predict the low bioavailability.

### Cowpea Acceptability

The second objective was to identify factors that influenced mothers to give cowpeas to their schoolchildren. We found that intention of mothers was strongly associated with and predicted behaviour. *Knowledge* of mothers about cowpeas and the *health values* they hold for their children were together associated with intention through *health behaviour identity* and *attitudes towards behaviour*. Knowledge and health values also predicted health behaviour identity. *Attitudes towards behaviour* and *perceived barriers* were the two internal constructs that predicted intention significantly albeit the predictive ability of the intention models was weak.

Other studies using the combined models of TPB and HBM [17], [23], [43] or the TPB alone [44], [45] have also shown strong association between intention and behaviour. The studies that measured behaviour and intention (cross-sectional or prospective) have also shown that intention is predictive of behaviour and may sometimes do so through an interaction with perceived barriers [23]. This seems to suggest that giving cowpeas to schoolchildren in northern Ghana may be largely driven by conscious efforts of mothers. It has however been shown that the proximity between the measurement of intention and behaviour can influence their association [46]; measured together (as in our case) can strengthen the association or, when behaviour is assessed after 5 weeks of measuring intention, weaken the association [47], [48]. However, in a related study in the same communities, frequency of consumption of cowpea-based meals at the household level was on average 2–3 times per week (Abizari, unpublished results), similar to values recorded here as intention and behaviour. As such, proximity may have had minimal influence on our measurements of intention and behaviour.

We did not observe a significant role of external factors (subjective norms, control beliefs and cues to action) on caregivers’ intention to give cowpeas to their schoolchild [23], [49]. This partly demonstrates that cowpeas are well accepted in the research area [13] and their consumption is not influenced by the opinion of health workers, husbands, mothers in-law and significant others. In line with the observations of Sheeran et al. [50], the absence of external influential factors would suggest that intention to give cowpeas is more likely to be stable if mothers have favourable attitudes. We doubt however whether if we had much younger reference children (<2 years) the outcome would have been the same. From our experience in the area, health workers encourage mothers of such children to give cowpeas to their children (especially when they are undernourished) ostensibly to improve iron status and growth. This may partly explain why we observed in this study that “child’s illness” is an important cue to give schoolchildren cowpeas. In our predictive model for behaviour we also observed age of child was a significant explanatory factor. It means that if iron-deficiency is presented in the context of illness, mothers would be more likely to accept a cowpea-based food promoted to contribute to reduce iron-deficiency.

Similar to the role of intention in the TPB model [15], the *health behaviour identity* was expected to mediate between “background and perception constructs” and *intention* in the combined model used in our study [17]. Our results confirm this mediating role of health behaviour identity [17], [23], [43]. In our work it means that the *knowledge* of mothers about cowpeas in combination with the *health values* they hold for their children made them have positive *health behaviour identity*. The positive *health behaviour identity* in turn yielded positive attitudes towards giving cowpeas to schoolchildren which subsequently predicted the intention of mothers to give cowpeas. It implies that if we reinforce mothers’ knowledge that “cowpeas give blood” and “support the growth of schoolchildren” coupled with the positive health values mothers hold for their children, it should be possible to promote cowpeas as likely vehicles to contribute to reduce iron-deficiency. Such a promotion may not be completely successful without addressing the barriers to behavioural intention. For instance, price of cowpea on the market was one of the barriers mentioned by caregivers. In their work Mishili et al. [51] reported that cowpea prices on the market start rising a few months after harvest. The implication is that rural households who have run out of cowpea stock may find it expensive to buy. Our results support the findings of Ndubuaku et al. [27] that abdominal discomforts, presence of weevils and long cooking time are barriers to cowpea consumption.

Internal reliability measures of our constructs were generally good and were within range of values observed by others [23], [43]. However, there is no prior indication of the reliability of the predictive models with regards to giving or intention to give cowpeas. Two other studies [23], [43] in Africa that utilized these predictive models have shown similar trend in low predictive abilities of the two models on intention. In our case the low predictive ability could be attributed to the generic reference to cowpeas rather than a specific cowpea-based food. In a study on iron-fortified soy sauce the predictive ability of the intention model was higher (15).

In summary what we have shown is that cowpea landraces from northern Ghana contain appreciable amounts of iron and zinc, but probably with a poor bioavailability. Attitudes towards giving cowpeas and perception of barriers are important predictors of mothers’ intention to give cowpeas to their schoolchildren. We have also shown that health behaviour identity may mediate but not predict intention of mothers. Finally our results suggest that knowledge about cowpeas and health values mothers hold for their children are key areas to focus attention in order to promote giving cowpeas to school children.
